# Exercise Therapy to Reduce Anxiety (ExTRA) in mid-life and later-life adults: pilot feasibility study

**DOI:** 10.1192/bjo.2026.12008

**Published:** 2026-06-08

**Authors:** Terence W. H. Chong, Andi Partovi, Julia McCurry, Eleanor Curran, David Ames, Kaarin J. Anstey, Alex Bahar-Fuchs, Christina Bryant, Kay L. Cox, Samantha M. Loi, Jenny Southam, Alissa Westphal, Nicola T. Lautenschlager

**Affiliations:** Department of Psychiatry, The University of Melbourne, Kew, Australia; Mental Health and Addiction Medicine Program, St Vincent’s Hospital Melbourne, Kew, Australia; Mental Health, The Royal Melbourne Hospital, Parkville, Australia; Department of Biomedical Engineering, The University of Melbourne, Parkville, Australia; School of Psychology, University of New South Wales, Sydney, Australia; Neuroscience Research Australia, Sydney, Australia; UNSW Ageing Futures Institute, University of New South Wales, Sydney, Australia; SEED Lifespan, School of Psychology, Deakin University, Burwood, Australia; Melbourne School of Psychological Sciences, The University of Melbourne, Parkville, Australia; Medical School, The University of Western Australia, Perth, Australia

**Keywords:** Anxiety, exercise, older adults, mid-life, technology

## Abstract

**Background:**

Anxiety is the most prevalent mental health condition, causing significant distress, disability and cost.

**Aims:**

This pilot study assessed the feasibility and acceptability of an eight-week self-directed online exercise intervention, supported by wearable activity monitors (WAMs), for mid-life and later-life adults with clinically significant anxiety, and provided preliminary estimates of effectiveness.

**Method:**

The Exercise Therapy to Reduce Anxiety (ExTRA) project recruited 21 participants aged 40–77 years with Depression, Anxiety and Stress Scales (DASS-21) anxiety scores >7. The ExTRA online platform integrated with the WAMs, and provided information about anxiety, exercise programmes and strategies to help meet national physical activity guidelines, overcome barriers and set goals.

**Results:**

ExTRA was feasible, with a 63% recruitment rate and 100% retention rate. Overall, evaluation questionnaire responses demonstrated high acceptability. At week 8, WAM average steps/day increased by 1358 (*p* = 0.036, Hedge’s *g* = −0.47), and fairly/very active minutes (3/6 metabolic equivalents) increased by 81.2 min/week (*p* = 0.024, *g* = −0.51), with moderate effect sizes. There was no statistically significant change on the self-report Global Physical Activity Questionnaire. The DASS-21 anxiety score reduction of 2.3 (*p* = 0.060, *g* = 0.42) from baseline to week 8 approached statistical significance. DASS-21 stress and depression scores were significantly reduced by 3.9 (*p* = 0.013, *g* = 0.57) and 3.7 (*p* = 0.02, *g* = 0.53), respectively, from baseline to week 8. There was no significant change in self-efficacy or dementia risk.

**Conclusions:**

The ExTRA intervention is feasible and acceptable, with positive signals for increasing objectively measured physical activity and improving mental health. Findings support and inform the design of randomised controlled trials to assess efficacy of this potentially automated and scalable intervention.

Anxiety and subthreshold anxiety disorders cause significant distress, disability and cost, yet remain relatively neglected in research.^1^ Anxiety disorders are also highly prevalent, with global prevalence estimated to be 10.6%, and in older adults ranging from 1.2 to 14%.^2^ Clinical practice guidelines for anxiety disorders, including those from the Royal Australian and New Zealand College of Psychiatrists and the UK National Institute for Health and Care Excellence recommend non-pharmacological interventions as part of holistic treatment.^3,4^

## Exercise interventions

There is emerging evidence that exercise interventions are helpful for anxiety disorders,^5–7^ in addition to their myriad benefits for mental and physical health and chronic disease prevention.^8^ Despite the benefits of exercise, 37% of Australian adults aged 18–64 years and 57% aged over 65 years did not meet the national physical activity guidelines in 2022.^9^ In Australia, similar to many developed nations, physical inactivity is the modifiable risk factor for dementia with the highest population attributable fraction of 8.3%.^10^ Mid-life and later-life are particularly critical periods for chronic disease prevention or intervention. Dementia risk reduction is increasingly a public health priority,^11^ and there is emerging evidence that anxiety may be a risk factor for developing dementia,^12,13^ although the Lancet Commission on dementia, intervention and care concluded that there was insufficient evidence.^14^ Physical inactivity is also a risk factor for heart disease, diabetes mellitus and some cancers.^15^

## Challenges and rationale for the Exercise Therapy to Reduce Anxiety project

One challenge of delivering exercise interventions for anxiety is that trials in populations with mental health issues have higher drop-out rates.^16^ This may be related to ‘exercise anxiety’ where an individual may interpret physical sensations related to exercise, such as sweating and breathlessness as being symptoms of anxiety.^17^ It is therefore important to tailor interventions to the needs of participants living with anxiety including information about anxiety and how to manage this when undertaking exercise.

A further challenge is to find ways to deliver exercise interventions to large populations in a cost-effective and scalable manner. Self-directed programmes with online resources and potentially automated feedback could be helpful where this mode is feasible and acceptable to users. Some individuals may still prefer directed and/or face-to-face interventions. Internet access and technology literacy in Australia continues to grow; however, there continues to be a ‘digital divide’, albeit reducing in size.^18^ Online exercise interventions have been shown to be feasible for older adults generally,^19^ but this is still limited, as a systematic review of online interventions for people with anxiety and depression only found three studies meeting inclusion criteria, with none including participants aged over 65 years and none superior to control.^20^

Qualitative interviews with 29 older adults who completed a physical activity trial found a general belief that physical activity interventions could reduce anxiety through increasing ‘mindfulness’ and/or through physiological benefits. In addition, participants expressed that ‘technology could help with information provision, health monitoring and motivation’, and ‘were open to using wearable activity monitors, online platforms and portable devices’.^21^ The use of wearable activity monitors (WAMs) in the general adult population has been demonstrated to be effective in a systematic review showing a moderate increase in physical activity equivalent to an additional 1235 daily steps and a small effect on increasing moderate to vigorous physical activity.^22^ In older adults, the use of WAMs has also been found to be associated with higher likelihood of meeting physical activity guidelines,^23^ as well as their use being acceptable to older adults.^24^

## Aims and hypotheses

The Exercise Therapy to Reduce Anxiety (ExTRA) project aimed to assess the feasibility and acceptability of an 8-week self-directed online exercise intervention, facilitated by the use of tailored strategies and WAMs, to reduce anxiety in mid-life and later-life adults with clinically significant anxiety. This study was the first that we are aware of that piloted a self-directed online exercise intervention designed by consumer, clinician and biomedical engineering researchers for this cohort that also included reminders and tailored advice with the potential to become automated in the future.

We also aimed to obtain preliminary data on changes to anxiety, physical activity level, self-efficacy, depression, stress and dementia risk to guide the design of a future randomised controlled trial.

Our hypothesis was that the intervention would be feasible as well as acceptable to participants. Although not powered to demonstrate this definitively, the study was designed to explore the benefits of the intervention in reducing anxiety and increasing physical activity. In addition, we also aimed to explore the benefits of the intervention on self-efficacy, depression, stress and dementia risk.

## Method

### Study design

This pilot feasibility and acceptability study utilised a pre–post experimental research design. The detailed study protocol was published previously.^25^ The authors assert that all procedures contributing to this work comply with the ethical standards of the relevant national and institutional committees on human experimentation and with the Helsinki Declaration of 1975, as revised in 2013. All procedures involving human participants were approved by The University of Melbourne Human Ethics Committee on 17 January 2023 (reference 2023-25560-37028-4). The study protocol was registered on the Australian New Zealand Clinical Trials Registry (registration number ACTRN12623000125628).

### Participants

Participants were recruited through advertising to the general public, health professionals and community organisations, including via emails, social media and a project website. The target sample size range was 20–30 participants, which is consistent with guidelines for pilot studies that suggest a sample size of 20–25 is likely to be sufficient to demonstrate efficacy in a single group with likely moderate or larger effect size,^26^ and a general ‘rule of thumb’ pilot study recommended sample size of 30 participants.^27^

Inclusion criteria were community dwelling adults aged between 40 and 79 years; with a Depression, Anxiety and Stress Scales (DASS-21) anxiety score greater than 7, which is a recognised cut-off for mild anxiety;^28^ with capacity to provide informed consent; access to the internet, email, smartphone and videoconference platforms; and the capacity to safely undertake an unsupervised moderate intensity exercise intervention, as determined by the research team with consultation with the participant’s general practitioner where needed. This means that participants have clinically significant anxiety, but do not need to have a diagnosis of an anxiety disorder, and may have other mental health symptoms and diagnoses provided this is not the focus of their presentation. The minimum age of 40 years is consistent with definitions of mid-life, which is a critical period of chronic disease prevention, whereas the upper age limit of 79 years was set because of the exercise intervention being unsupervised and the increased frailty and risk associated with exercise with older age.

Exclusion criteria included insufficient English literacy to complete outcome measures, significant medical conditions precluding participation in an unsupervised moderate intensity exercise intervention, or the focus of their presentation being another mental health symptom such as psychosis or mania, or acute risk such as suicidality. The inclusion and exclusion criteria relating to diagnosis and comorbid conditions were intentionally broad to be inclusive as this study aimed to be ‘real world’ and pragmatic, especially as benefits of exercise have been shown to be effective across many mental health conditions.

After providing informed consent online via electronic signature and completing an online screening questionnaire including the DASS-21, participants were then assessed by a psychiatrist clinician researcher via videoconference to confirm that inclusion criteria were met. This assessment included diagnostic and mental health risk clarification, and consideration of capacity to safely undertake a moderate intensity exercise programme such as walking.

### Intervention

**Fig. 1:**
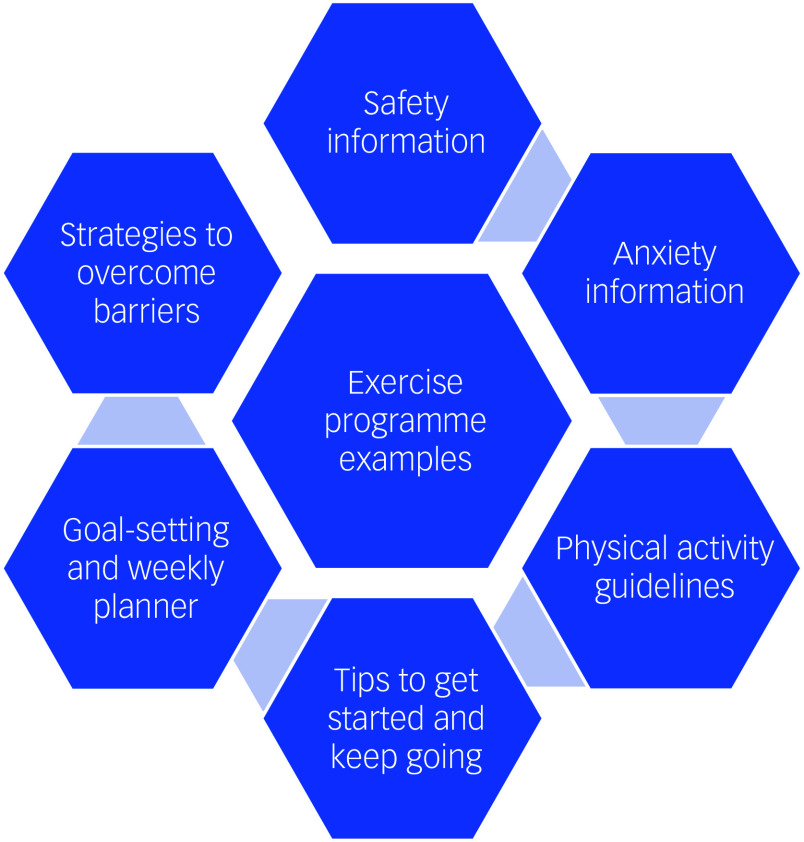
Exercise Therapy to Reduce Anxiety (ExTRA) online platform information.

The intervention was delivered via the ExTRA online platform. This platform provided information in the form of text, images, videos and links to resources, and this content is summarised in Fig. 1. The information included psychoeducation about anxiety and exercising while living with anxiety. This was followed by information about exercise, including the national physical activity guideline recommendations for the relevant age groups.^29–31^ Resources included those adapted from the team’s Exercise for Cognitive Health (EXCEL) project, a trial of a researcher ‘coach’ remotely delivered exercise intervention for middle-aged and older participants with memory complaints and anxiety, depression and/or stress.^32^ These resources included safety information, tips to get started and keep going, warming up and cooling down, detailed information on aerobic exercise focusing on walking, goal-setting tools, weekly planners, example walking programmes and strategies to overcome common barriers to physical activity, including anxiety.

**Fig. 2:**
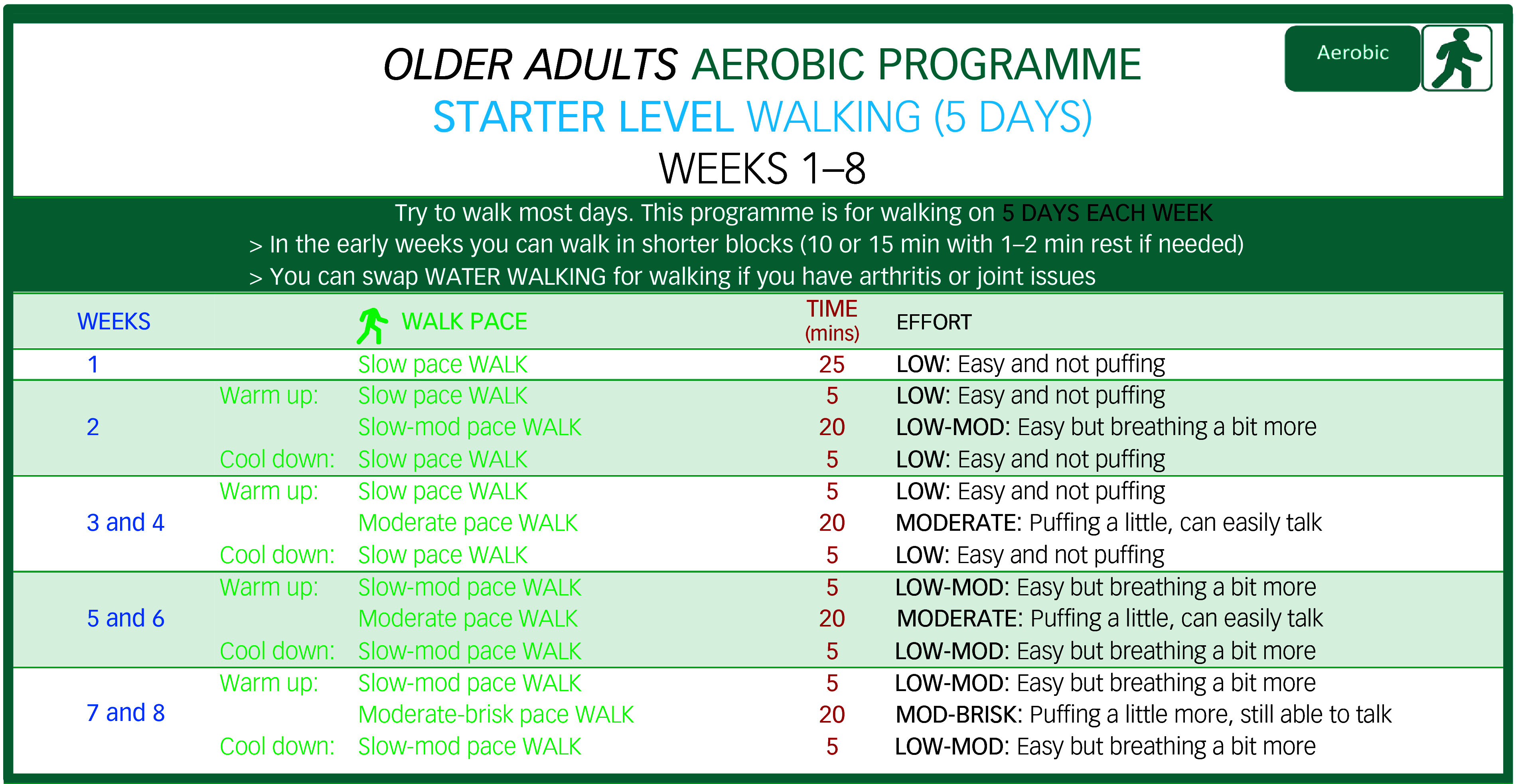
Example of exercise programme.

The intervention resources were used to support participants to gradually work toward meeting the physical activity guideline recommendation of moderate intensity aerobic physical activity for at least 150 min per week or 30 min on most days, during the 8-week programme. If a participant was already meeting the guidelines, they were encouraged to increase their aerobic physical activity either through longer duration or higher intensity. The example programmes involved walking as this was considered a safe and accessible type of exercise. Figure 2 shows the example programme provided to adults aged 65–79 years at a starter level wanting to exercise 5 days per week. There were also example programmes for adults aged 40–64 years, intermediate level programmes for participants who were already active and programmes for participants wanting to exercise 3 days per week. The resources also provided guidance to participants that they could approximately gauge moderate intensity aerobic activity as when they ‘start to sweat and need to breathe harder but can still talk in short sentences’, with examples being brisk walking or cycling. Vigorous intensity aerobic activity was defined as ‘causing sweating and feeling out of breath so that it would be hard to hold a conversation’, with examples being running, faster swimming and aerobics in the gym. Given that the programme was self-directed, participants were able to choose to follow the example programmes or to adapt them to their own situation and preferences; for example, changing the duration or frequency of exercise or changing the type of exercise from walking to an aerobic alternative such as cycling or swimming, while still working toward the aim of meeting the guidelines.

Participants were given a WAM (Fitbit Inspire 2) and provided with written instructions on its setup and also a videoconference meeting if needed. They were asked to wear the Fitbit all day for the duration of the trial, but could take it off when asleep if they preferred. Participants could keep the Fitbit after they finished the project to help them to continue their exercise. Fitbit step data was also integrated with the ExTRA online platform so that step data could also be viewed by participants online. Each week, participants received an encouragement email from the research team that reported their WAM step count, reminded them to continue their activity and requested that they complete the Barriers Self-Efficacy Scale (BARSE).^33^ The email also included strategies to overcome a selected common barrier to exercise. The selected strategy in the weekly email was based on an algorithm that could be automated in future. This algorithm selected the barrier from the BARSE scale that the participant had reported the least confidence in overcoming. If that strategy had been previously sent in an email, then the next most difficult barrier was selected, whereas if all barriers were rated as equally difficult, then the selected strategy would just address the next barrier on the list. Although not necessarily a real-world strategy, this email could potentially become an automated part of the online platform in future given that it followed a defined template and algorithm.

The ExTRA platform also incorporated behaviour change strategies informed by qualitative interviews with completers of a previous physical activity randomised controlled trial (INDIGO), which identified the themes of structure and accountability, self-efficacy, habit formation and enjoyment.^34^ These strategies were further informed by intervention components suggested in a model for physical activity intervention tailoring developed from qualitative research with participants experiencing cognitive concerns and symptoms of depression and anxiety^35^ and an existing behavioural science model called the Capability, Opportunity and Motivation System of Behaviour model.^36^ The specific components incorporated into the ExTRA platform included presentation of information about benefits of physical activity for anxiety; provision of lay-person versions of physical activity guidelines, including examples of required intensity; links to trusted expert sources; goal-setting and weekly planner tools; example graded physical activity programmes designed by exercise specialists; provision of WAMs that reported daily steps and printable format of all online platform information to enable hard-copy option where preferred.^35^ Furthermore, as a self-directed programme, participants were shown the resources, but were able to choose which they would use, as research has shown that preferences for tools vary significantly between trial participants.^34^

### Outcomes

Collected outcome measures and time points are summarised in Table 1. A screening videoconference meeting confirmed informed consent and checked inclusion and exclusion criteria, including the DASS-21 scale score. During the week before commencing the intervention, baseline outcome measures were completed. At the conclusion of the 8-week intervention, outcome measures were reassessed. Study data was collected and managed using REDCap (version 16.0.22 for Windows; Vanderbilt University, Nashville, Tennessee, USA; https://projectredcap.org/) electronic data capture tools hosted at The University of Melbourne, and integrated into the ExTRA online platform.

**Table 1:** Summary of outcome measures and time points

Outcome	Measure	Time point									
		Screening	Baseline Week –1	Week 1	Week 2	Week 3	Week 4	Week 5	Week 6	Week 7	Completion Week 8
Physical activity level (WAM)	Steps/day Fairly and very active mins/week		x	x	x	x	x	x	x	x	x
Physical activity level (self-report)	GPAQ WHO		x				x				x
Anxiety, stress and depression	DASS-21	x	x								x
Exercise self-efficacy	EXSE		x				x				x
Barriers to exercise self-efficacy	BARSE		x	x	x	x	x	x	x	x	x
Dementia risk	ANU-ADRI-SF		x								x
Acceptability	Evaluation questionnaire										x

WAM, wearable activity monitor; GPAQ WHO, Global Physical Activity Questionnaire World Health Organization; DASS-21, Depression, Anxiety and Stress Scales 21; EXSE, Exercise Self-efficacy Scale; BARSE, Barriers Self-efficacy Scale; ANU-ADRI-SF, Australian National University Alzheimer’s Disease Risk Index Short Form.

### Primary outcomes

Feasibility was measured by

- target screening rate (proportion of potential participants who meet study inclusion criteria out of participants who completed the screening questionnaire) of at least 70% as per protocol;^25^
- target retention rate (proportion of participants who completed week 8 follow-up outcome measures out of participants who commenced the intervention) of at least 65% as per protocol.^25^

Acceptability and usability were measured using an evaluation questionnaire. The latter employed qualitative questions and Likert scale (range 0–5) questions (mean score of at least three) regarding programme helpfulness and enjoyment. The questions were prepared by the research team to obtain data on participants’ views of acceptability and helpfulness of the programme, its various components and mode of delivery. Apart from providing information about acceptability, the data will also help with improving the programme for future research trials. Website usage data outcomes as per protocol^25^ were not available from the platform.

Physical activity level was measured using the WAM (worn throughout the study) and the Global Physical Activity Questionnaire (GPAQ)^37^ (completed at baseline and weeks 4 and 8). WAM data included steps per day, minutes of lightly, fairly active (three metabolic equivalents) and very active (six metabolic equivalents) physical activity minutes per week and sedentary minutes per day. These measures were collected using the Fitbit Inspire 2, which calculates this data using their proprietary algorithm.^38^ Although these data were collected throughout the duration of the intervention, the mean scores for the week before the intervention commencing (baseline) and week 8 (completion) were used in the analysis. Where the WAM recorded zero daily steps, indicating that the WAM was either not worn or the battery was flat (only two occurrences during the baseline or completion weeks), that day was excluded and the mean for that week was taken across the other 6 days of collected data. The GPAQ is a 16-item questionnaire about physical activity that provided self-report data including minutes/week of moderate intensity (four metabolic equivalents) and vigorous intensity (eight metabolic equivalents) physical activity.^37^

Anxiety was measured by completing the DASS-21 online (screening, baseline and week 8), which is a 21-item self-report tool with seven items measuring each of depression, anxiety and stress. Each item was rated on a four-point severity scale from 0 to 3 points, resulting in subscale scores that ranged from 0 to 42 points.^28^

### Secondary outcomes

Depression and stress were measured by the relevant DASS-21 subscales at screening, baseline and week 8.

Self-efficacy was self-reported using the Exercise Self-efficacy Scale (EXSE)^39^ (baseline, weeks 4 and 8) and BARSE^33^ (baseline, weeks 1–8). The EXSE measured self-efficacy to undertake exercise, with eight items scored from 0% (not at all confident) to 100% (highly confident) in 10% intervals, with the overall score being the average of all items.^39^ The BARSE measured self-efficacy to overcome barriers to engaging in exercise, with 13 items scored the same as the EXSE.^33^ Both the BARSE and EXSE are validated self-efficacy scales and have been used in exercise intervention trials.^33,39^

Dementia risk was measured using the self-report ANU-Alzheimer’s Disease Risk Index Short Form (ANU-ADRI-SF)^40^ (baseline and week 8). This is a self-report questionnaire-based dementia risk assessment tool that takes around 5 min to complete and is a validated shorter version of the original ANU-ADRI tool, which takes around 15–20 min.^40^ The tool addresses 11 risk and four protective factors for Alzheimer’s disease.^40^ Unfortunately, a REDCap technical error meant the minutes/day walking question (Q25) was not displayed to participants. A GPAQ question (question 9) about minutes/day walking or cycling for commuting, although not identical, functioned as a substitution.

### Data analysis

Descriptive statistics were used to describe the demographic and clinical characteristics of participants as well as the outcome measures collected. Free-text data collected in the evaluation questionnaire were summarised narratively. Paired-samples *t*-tests were used to compare outcomes between baseline and completion as well as screening and completion. Estimates of effect sizes were calculated using Cohen’s *d* with the Hedges’ correction for small sample sizes. No controlling for testing multiple hypotheses was performed. Statistical analysis was conducted using IBM SPSS Statistics Version 30 for Windows (IBM Corp., Armonk, New York, USA; https://www.ibm.com/products/spss-statistics). Given the small sample size, data was checked manually by the research team and outlier answers checked with participants to confirm their accuracy. Apart from the previously mentioned two occurrences of missing WAM data, there was no other missing data.

## Results

### Participant characteristics

Demographic and clinical characteristics of participants (*N* = 21) are reported in Table 2. Mean age of participants was 53.7 years with a range from 40 to 77 years, and approximately three-quarters were women. Participants were generally highly educated and approximately two-thirds were in a relationship. Thirteen participants reported diagnosis of an anxiety disorder, ten reported a depressive disorder, four reported post-traumatic stress disorder, one reported attention-deficit hyperactivity disorder, eating disorder and obsessive–compulsive disorder, and six reported no diagnosis. Approximately a quarter of participants were taking psychotropic medication.

**Table 2:** Demographic and clinical characteristics of participants at baseline (*N* = 21)

Characteristic (continuous variables)	Mean (s.d.)	Median	Range
Age (years)	53.7 (10.9)	51.5	40.5–77.5
Education (years)	17.9 (4.5)	19	8–27
Body mass index (kg/m^2^)	26.2 (5.2)	24.3	19.8–40.4

Characteristic (categorical variables)	*n* (%)		
Gender			
Female	16 (76.2)		
Male	5 (23.8)		
Marital status			
Married	10 (47.6)		
*De facto*	4 (19.0)		
Separated	1 (4.8)		
Divorced	1 (4.8)		
Widowed	0 (0.0)		
Never married	5 (23.8)		
Mental health diagnoses			
None	6 (28.6)		
Attention-deficit hyperactivity disorder	1 (4.8)		
Anxiety disorders	13 (61.9)		
Depressive disorders	10 (47.6)		
Eating disorders	1 (4.8)		
Obsessive–compulsive disorder	1 (4.8)		
Post-traumatic stress disorder	4 (19.0)		
Taking psychotropic medication	6 (28.6)		

### Feasibility and acceptability

**Fig. 3:**
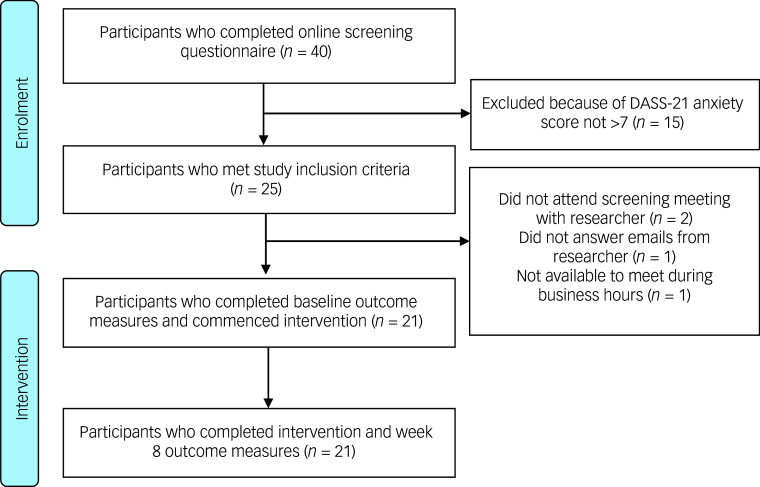
Participant flow through the project. DASS-21, Depression, Anxiety and Stress Scales 21.

Participant flow through the project is reported in Fig. 3. The screening rate (proportion of potential participants who met study inclusion criteria out of participants who completed the screening questionnaire) was 63%, which was below the target of 70%. All 15 participant exclusions were attributable to low DASS-21 anxiety subscale scores not being above 7. The retention rate (proportion of participants who completed week 8 follow-up outcome measures out of participants who commenced the intervention) was 100%, which was well above the target of 65%. In addition, the baseline and week 8 outcome measures completion rate was 100%, and the weekly outcome measures completion rate was 86%, which demonstrated regular engagement with the online platform.

Overall, the ExTRA intervention received positive feedback from participants in the evaluation questionnaire, with results summarised in Table 3. Almost all participants enjoyed their involvement (95%) and found it useful (86%), and two-thirds reported that it was helpful for their anxiety. Most would continue the programme (86%), and the majority preferred online delivery (62%) or combination (29%), with only two participants preferring an in-person approach (10%). All participants found the Fitbit easy to use and most did not have problems with using it or the online platform. Mean scores on the Likert scales about enjoyment and usefulness of the online platform website, WAM and weekly email were all above 3. Free-text answers to questions in the evaluation questionnaire were also very helpful to inform future trial design.

**Table 3:** Evaluation questionnaire results summary (*N* = 21)

Question	*n* (%) or *Mean (s.d.)
Have you enjoyed your involvement in the ExTRA study over the 8 weeks?	
Yes	20 (95.2)
No	1 (4.8)
How enjoyable did you find the (physical activity) programme? (Likert scale 1)	
*Mean (s.d.)	3.7 (1.2)*
How helpful did you find the following elements of your physical activity programme? (Likert scale 2)	
The physical activity information provided on the ExTRA website	
*Mean (s.d.)	3.5 (1.1)*
The WAM (Fitbit)	
*Mean (s.d.)	4.6 (0.8)*
The weekly email with links to helpful resources	
*Mean (s.d.)	3.6 (1.5)*
After being in the study for 8 weeks, do you think the intervention was useful?	
Yes	18 (85.7)
No	3 (14.3)
Do you think the programme has changed your anxiety level during the 8 weeks?	
Improved	14 (66.7)
No change	7 (33.3)
Worsened	0 (0.0)
Do you think you will continue the physical activity programme after the 8 weeks of this project?	
Yes	18 (85.7)
No	3 (14.3)
What format would you prefer the ExTRA programme to be delivered through?	
Online	13 (61.9)
In person	2 (9.5)
Mixture of both	6 (28.6)
How often did you encounter problems with the ExTRA website?	
Never	16 (76.2)
Seldom	3 (14.3)
Sometimes	2 (9.5)
Frequently	0 (0.0)
Always	0 (0.0)
Did you find the WAM (Fitbit) easy to use?	
Yes	21 (100.0)
No	0 (0.0)
How often did you encounter problems with the WAM (Fitbit)?	
Never	15 (71.4)
Seldom	4 (19.0)
Sometimes	0 (0.0)
Frequently	2 (9.5)
Always	0 (0.0)

ExTRA, Exercise Therapy to Reduce Anxiety; WAM, wearable activity monitor.

Likert scale 1: Did not undertake = 0, Not enjoyable = 1, Somewhat enjoyable = 2, Enjoyable = 3, Very enjoyable = 4, Extremely enjoyable = 5.

Likert scale 2: Did not apply = 0, Very unhelpful = 1, Unhelpful = 2, Neutral = 3, Helpful = 4, Very helpful = 5.

### Outcome measures

At baseline, approximately a third of participants were not completing 150 min of at least moderate intensity aerobic physical activity per week. Of note, the DASS-21 scores at baseline were all lower than at screening. The mean DASS-21 anxiety score was 16.6 (severe) at screening and 9.9 (moderate) at baseline. For DASS-21 stress, this was 25.2 (moderate) and 19.0 (moderate), and DASS-21 depression was 19.1 (moderate) and 12.5 (mild), at screening and baseline, respectively. The mean time between the screening and baseline was 16.8 days, which included the time taken for delivery of the WAM to participants and to organise the screening and baseline meetings with participants. On average, participants were about 70% confident on the EXSE and about 55% confident in overcoming barriers to exercise at baseline on the BARSE.

Paired-samples *t*-tests were used to compare changes in outcome measures from baseline to week 8, with results summarised in Table 4. The changes in the DASS-21 were also compared from screening to week 8, along with some exploratory subgroup analyses with these results reported in Table 5. There were statistically significant baseline to week 8 increases in physical activity measured by the WAM, with a mean increase of 1358 steps per day and 81 min per week of fairly (three metabolic equivalents) and very (six metabolic equivalents) active minutes per week. This was not the case using the self-report GPAQ, albeit with different definitions of moderate (four metabolic equivalents) and vigorous (eight metabolic equivalents) intensity.

**Table 4:** Change in outcome measures from baseline to week 8

Outcome measure	Baseline Mean (s.d.)	Week 8 Mean (s.d.)	Change Mean (s.d.)	*p*-value^a^	Effect size Cohen’s *d* (95% CI)^b^
Steps per day (Fitbit)	9199 (4554)	10 558 (4264)	1358 (2769)^^*^^	0.036	−0.47 (−0.90 to 0.03)
Fairly and very active time (mins/week) (Fitbit)	266.0 (243.9)	347.2 (267.9)	81.2 (153.0)^^*^^	0.024	−0.51 (−0.95 to 0.07)
Moderate and vigorous physical activity (mins/week) (GPAQ)	347.9 (447.0)	383.8 (390.3)	36.0 (280.4)	0.56	−0.12 (−0.54 to 0.29)
Anxiety (DASS-21)	9.9 (7.1)	7.6 (6.2)	−2.3 (5.3)	0.060	0.42 (−0.18 to 0.84)
Stress (DASS-21)	19.0 (6.9)	15.1 (10.7)	−3.9 (6.6)^^*^^	0.013	0.57 (0.12 to 1.01)
Depression (DASS-21)	12.5 (9.7)	8.8 (9.5)	−3.7 (6.7)^^*^^	0.020	0.53 (0.08 to 0.97)
Exercise self-efficacy (EXSE)	70.0 (23.7)	64.8 (26.2)	−5.2 (24.8)	0.35	0.20 (−0.22 to 0.62)
Barriers to self-efficacy (BARSE)	55.1 (22.9)	62.7 (25.1)	7.6 (16.8)	0.052	−0.43 (−0.86 to 0.004)
Dementia risk (ANU-ADRI-SF)	0.29 (7.3)	−0.095 (7.7)	−0.38 (2.5)	0.50	0.14 (−0.27 to 0.56)

GPAQ, Global Physical Activity Questionnaire; DASS-21, Depression, Anxiety and Stress Scales 21; EXSE, Exercise Self-efficacy Scale; BARSE, Barriers Self-efficacy Scale; ANU-ADRI-SF, Australian National University Alzheimer’s Disease Risk Index Short Form.

* *p* < 0.05.

a. Two-sided *p*-value.

b. Cohen’s *d* with Hedges’ correction.

**Table 5:** Exploratory analyses

Measure (DASS-21)	Screening Mean (s.d.)	Week 8 Mean (s.d.)	Change Mean (s.d.)	*p*-value^a^	Effect size Cohen’s *d* (95% CI)^b^
Anxiety	16.6 (6.2)	7.6 (6.2)	−9.0 (7.8)^^*^^	<0.001	1.11 (0.57–1.64)
Stress	25.3 (5.3)	15.1 (10.7)	−10.2 (8.2)^^*^^	<0.001	1.19 (0.63–1.74)
Depression	19.1 (11.1)	8.8 (9.5)	−10.4 (7.4)^^*^^	<0.001	1.34 (0.75–1.92)

Outcome measure	Baseline Mean (s.d.)	Week 8 Mean (s.d.)	Change Mean (s.d.)	*p*-value^a^	Effect size Cohen’s *d* (CI)^b^
Subgroup with DASS-21 anxiety score still >7 at baseline (*n* = 13)					
Anxiety (DASS-21)	14.3 (5.3)	10.3 (6.4)	−4.0 (6.1)^^*^^	0.035	0.62 (0.04–1.2)
Subgroup with <150 min/week of fairly or very active physical activity (Fitbit) (*n* = 7)					
Steps per day (Fitbit)	6889 (2475)	8418 (2831)	1528 (2401)	0.14	−0.55 (−1.25 to 0.18)
Fairly and very active time (mins/week) (Fitbit)	43.1 (30.2)	149.1 (88.5)	106.0 (73.0)^^*^^	0.009	−1.26 (−2.19 to 0.29)
Anxiety (DASS-21)	11.1 (7.5)	8.0 (4.9)	−3.1 (4.7)	0.13	0.58 (−0.16 to 1.3)
Stress (DASS-21)	15.1 (4.3)	10.9 (4.0)	−4.3 (4.8)	0.057	0.77 (−0.02 to 1.5)
Depression (DASS-21)	14.6 (10.6)	10.6 (12.4)	−4.0 (8.4)	0.26	0.41 (−0.28 to 1.1)
Subgroup with ≥150 min/week of fairly or very active physical activity (Fitbit) (*n* = 14)					
Steps per day (Fitbit)	10 355 (4977)	11 627 (4537)	1273 (3019)	0.14	−0.40 (−0.91 to 0.13)
Fairly and very active time (min/week) (Fitbit)	377.4 (225.7)	446.3 (274.1)	68.9 (181.8)	0.18	−0.36 (−0.86 to 0.16)
Anxiety (DASS-21)	9.3 (7.1)	7.4 (6.9)	−1.9 (5.6)	0.24	0.31 (−0.20 to 0.81)
Stress (DASS-21)	21.0 (7.2)	17.3 (12.4)	−3.7 (7.4)	0.084	0.47 (−0.06 to 0.99)
Depression (DASS-21)	11.4 (9.5)	7.9 (8.1)	−3.6 (6.0)^^*^^	0.045	0.56 (0.01 to 1.08)

DASS-21, Depression, Anxiety and Stress Scales 21.

* *p* < 0.05.

a. Two-sided *p*-value.

b. Cohen’s *d* with Hedges’ correction.

DASS-21 anxiety reduced by 2.3 points from baseline to week 8, which was approaching statistical significance (*p* = 0.060), and 9.0 points from screening to week 8, which was statistically significant (*p* < 0.001). However, when the scores of the subgroup of 13 participants that still met the criterion of DASS-21 anxiety score greater than 7 at baseline were analysed, their 4.0 point reduction from baseline to week 8 was statistically significant (*p* = 0.035). DASS-21 stress and depression score reductions were statistically significant, with the same pattern of a larger reduction from screening to week 8 compared with baseline to week 8. The improvement in self-efficacy to overcome barriers to exercise also improved from baseline to week 8 was also approaching statistical significance. Cohen’s *d* with the Hedges’ correction was used to estimate effect sizes. Most of the effect sizes were medium, although they were large for the DASS-21 changes from screening to week 8. When investigating the outcomes of the subgroups of participants meeting and not meeting the guidelines of 150 min of fairly or very active minutes of physical activity per week, the results become much less conclusive, which may reflect the small sizes of the subgroups. One difference that was observed was that the subgroup not meeting the aerobic physical activity guidelines had a statistically significant increase of 106 min of fairly and very active minutes of per week.

## Discussion

The aim of this pilot study was to assess the feasibility and acceptability of the ExTRA intervention and obtain preliminary estimates of its potential efficacy on physical activity and anxiety levels. Given the accepted general benefits of exercise on physical, mental and cognitive health, particularly in studies with more researcher involvement, this study focused on a real-world setting with deliberately broad transdiagnostic inclusion criteria, and a delivery model that is scalable and has potential for automation in a fully powered randomised controlled trial.

Overall feasibility results were positive with the ExTRA intervention’s screening (63%), retention (100%) and outcome measure completion (100% baseline/week 8 and 86% weekly surveys) rates either having met or being close to meeting targets. The screening rate was below the 70% target, with all exclusions due to not meeting the inclusion criterion of a DASS-21 anxiety score greater than 7. This could reflect potential participants misrecognising other symptoms such as stress or depression, as anxiety, or an intrinsic issue with the scale itself, particularly for older adult cohorts. In older adults, the DASS-21 anxiety subscale has been reported to have lower internal consistency, but still practical to use in lieu of multiple scales to reduce participant burden.^41^ Consideration could be given to adding an older adult specific scale such as the Geriatric Anxiety Inventory in a future randomised controlled trial.

The ExTRA intervention was also shown to be acceptable to participants, with almost all participants reporting that they enjoyed being in the study, found it useful and planned to continue with exercise post-study, as well as two-thirds of participants reporting that their anxiety had subjectively improved. The majority of participants preferred completely online or a combination of online and in-person delivery. The challenge with in-person delivery would be about scalability and access, so it was promising to see that the completely online option would be preferred by the majority of participants. It does mean that programmes may need to consider a more resource-intensive in-person option for some participants. Most participants found the WAM helpful, whereas the weekly email and the information resources on the ExTRA platform were a bit more mixed, with over half of participants finding them helpful. The free-text responses in the evaluation questionnaire will be valuable in guiding intervention refinement for a future randomised controlled trial.

From baseline to week 8, there were statistically significant increases in physical activity, with both daily steps and very (six metabolic equivalents) active minutes of physical activity per week increasing with moderate effect sizes. However, this was not seen in the self-report GPAQ data for moderate (four metabolic equivalents) and vigorous (eight metabolic equivalents) intensity physical activity per week. The findings were either similar or superior to those described in a systematic review of studies that used WAMs in 18- to 65-year-old participants to increase physical activity for any indication, which found moderate effects on steps equivalent to 1235 daily steps and a small effect on moderate to vigorous physical activity equivalent to 48.5 min per week.^22^ This result is particularly promising given that physical inactivity is the modifiable dementia risk factor with the largest population attributable fraction in Australia, and many Australian adults and older adults do not meet national physical activity guidelines.

The DASS-21 anxiety, stress and depression subscale scores showed reductions from baseline to week 8 that were statistically significant for stress and depression, but only approached statistical significance for anxiety, all with moderate effect sizes. These findings demonstrated some similarity with those found in previous systematic reviews of exercise interventions for anxiety that were not specific to older adults and mostly not delivered online,^5,7^ and also a review including mid-life and older women.^42^ In addition, our findings are consistent with the existing evidence supporting the efficacy of exercise interventions, which is more established for depression compared with stress,^43^ which means that the intervention may potentially have broader mental health benefits than anxiety alone. There was also an improvement in self-efficacy to overcome barriers to exercise that was approaching statistical significance.

When we undertook a subgroup analysis of the 13 participants who still met the DASS-21 anxiety score of greater than 7 at baseline, the reduction in DASS-21 score from baseline to completion became statistically significant. This suggested that there needed to be at least a mild level of anxiety on the DASS-21 anxiety subscale to be able to demonstrate an improvement. This finding could have implications for the definition of the inclusion criteria for a future randomised controlled trial. There was also the suggestion that participants who were not meeting aerobic physical activity guidelines may benefit more than participants who were not, although the study was not powered to investigate this. This may suggest that future studies could focus on including participants who are not meeting the physical activity guidelines.

The changes in DASS-21 scores from screening to week 8 were all statistically significant with large effect sizes. The improvement in the DASS-21 scores between screening and baseline could be explained by the presence of a beneficial effect from enrolling in the study, possibly related to a sense of mastery or control, the real-world nature of the study meaning that participants could be receiving other treatments, or variation in scores over time. The trend toward reduction in scores across anxiety, stress and depression somewhat reduced the likelihood of the latter explanation. The placebo effect is also a well-recognised phenomenon in medicine and psychiatry.^44^ This finding emphasised the importance of having a control group in a future study. Despite this, the DASS-21 scores continued to improve between baseline and week 8, although only approaching statistical significance for the anxiety subscale. This still supported the potential for the ExTRA intervention to be feasible and scalable. The screening rate was just under the target, and all exclusions occurred as a result of potential participants not scoring greater than 7 on the DASS-21 anxiety scale. This could suggest that the recruitment information was not sufficiently clear, potential participants were misinterpreting other symptoms as anxiety, or perhaps that a scale designed for older adults such as the Geriatric Anxiety Inventory might be more useful for participants over 65 years of age. Measures of function and quality of life could also add more helpful information about participants in a future trial.

Limitations of the study included that there were more female participants than male, participants on average had high levels of education, and some were already quite physically active. The reduction in DASS-21 scores between screening and baseline was also a limitation and may reflect the recognised placebo effect that can occur in clinical trials. This emphasises the importance of the control group in a future randomised controlled trial. Other limitations were the relatively short follow-up at 8 weeks, need for participants to have sufficient English language skills to complete outcome measures, and also have access to technology and be able to use this. An additional limitation was the decision not to control for testing multiple hypotheses because of the pilot exploratory nature of the trial focusing on feasibility and acceptability rather than efficacy. This could be addressed in a future efficacy trial by the use of techniques such as a Bonferroni correction or similar, depending on the study design employed. Nevertheless, the aim of the study was to pilot a potentially scalable and automated intervention, while also acknowledging that there may be specific groups in the population that still would require specific intervention modalities and increased support.

The significant strength of this study was the pilot of what the authors believe to be the first feasible and acceptable online self-directed exercise intervention, facilitated by a WAM, for mid-life and later-life adults with clinically significant anxiety in a real-world setting. Further strengths are that the ExTRA intervention adapted resources from other effective interventions that were used in more resource-intensive trials,^32^ and also was informed by key qualitative findings from previous studies.^21,34^ The broad inclusion criteria with few exclusion criteria, transdiagnostic approach and use of objective physical activity outcome measures are other key strengths.

To summarise, anxiety is the most prevalent mental health condition and causes significant distress, disability and cost, yet effective non-pharmacological interventions for it remain relatively neglected by researchers and clinicians. Non-pharmacological interventions are recommended by guidelines as part of holistic treatment for anxiety and exercise has emerging evidence of effectiveness. Given the myriad benefits of exercise for health and its role in chronic disease prevention, including dementia, mid-life and later-life are critical periods for intervention. Resource-intensive interventions have been shown to be helpful, but there is a need for scalable interventions to be able to reach large populations. The feasibility, acceptability and estimates of effectiveness found in this pilot study both inform the design and provide key support for a fully powered, randomised controlled trial to evaluate the clinical and cost effectiveness of the ExTRA intervention for mid-life and later-life adults with clinically significant anxiety.

## Data Availability

The data that support the findings of this study are available on request from the corresponding author, T.W.H.C.
